# Familial associations of lymphoma and myeloma with autoimmune diseases

**DOI:** 10.1038/bcj.2016.123

**Published:** 2017-01-06

**Authors:** K Hemminki, A Försti, K Sundquist, J Sundquist, X Li

**Affiliations:** 1 Division of Molecular Genetic Epidemiology, German Cancer Research Center (DKFZ), Heidelberg, Germany; 2 Center for Primary Health Care Research, Lund University, Malmö, Sweden; 3 Stanford Prevention Research Center, Stanford University School of Medicine, Stanford, CA, USA

## Abstract

Many B-cell neoplasms are associated with autoimmune diseases (AIDs) but most evidence is based on a personal rather than a family history of AIDs. Here we calculated risks for non-Hodgkin lymphoma (NHL), Hodgkin lymphoma (HL) and multiple myeloma (MM) when family members were diagnosed with any of 44 different AIDs, or, independently, risk for AIDs when family members were diagnosed with a neoplasm. A total of 64 418 neoplasms and 531 155 AIDs were identified from Swedish nationwide health care records. NHL was associated with a family history of five AIDs, all increasing the risk, HL was associated with one AID increasing and three AIDs decreasing the risk while MM had no association. A family history of NHL was associated with eight, HL with seven and MM with seven different AIDs, nine increasing and 13 decreasing the risk. The present family data on B-cell neoplasms and AIDs show an approximately equal number of associations for risk increase and risk decrease, suggesting that inherited genes or gene-environment interactions may increase the risk or be protective. These results differed from published data on personal history of AID, which only report increased risks, often vastly higher and for different AIDs compared with the present data.

## Introduction

Hodgkin lymphoma (HL), multiple myeloma (MM) and the common non-Hodgkin lymphoma (NHL) subtypes are mature B-cell neoplasms for which a personal history of an autoimmune disease (AID) is a known risk factor.^1, 2, 3, 4, 5, 6, 7, 8^ AIDs are characterized by loss of tolerance toward own antigens and destruction of body's own constituents.^9, 10^ AIDs are a heterogeneous group of diseases, either with a systemic or a localized presentation and with or without circulating autoantibodies; the prevalence ranges from very rare to moderately common, jointly reaching a population prevalence of up to 10%.^11^ Data from the literature show that diverse AIDs appear to be associated with diverse neoplasms, including different B-cell neoplasms, and no mechanistic bases for the selective response have been demonstrated. In a recent study, we provided evidence that germinal center-derived neoplasms were most responsive to autoimmune stimulation and that these neoplasms showed patterns of similarity in their response to different AIDs.^8, 12^

Even though a personal history of an AID is a known risk factor for B-cell neoplasms the data on associations of a family history of AID with cancer risk has deserved limited attention although the possible genetic etiology calls for clarification. In Swedish–Danish studies, a family history of sarcoidosis and ulcerative colitis was reported to be associated with an increased risk of HL, whereas for NHL no significant association with AIDs was found.^13, 14^ For MM, a family history of systemic lupus erythematosus was a risk factor.^15^ The potential mechanisms for shared familial associations could be pleiotropy, that is, single genes influencing multiple phenotypes, such as different cancers or AIDs.^16, 17, 18, 19^ Preliminary evidence for pleiotropy between B-cell neoplasms and many AIDs has been presented for human leukocyte antigen alleles and some other genes.^20, 21, 22^ The intriguing aspect of pleiotropy is that the direction of allelic effects may be opposite between the two diseases.^16, 17, 18, 19^

In the present study, we will analyze risks for NHL, HL and MM when a first-degree family member was diagnosed with any of 44 AIDs, and vice versa, risks for individual AIDs when a family member was diagnosed with a B-cell neoplasm. A total of 64 418 neoplasms and 531 155 AIDs were covered and all diagnoses were obtained from nationwide medical records.

## Methods

AID patients were identified from the Swedish Hospital Discharge Register (years 1964 through 2012, full national coverage from 1986 onwards) and the Outpatient Register (2001 through 2012). Family relationships were obtained from the Multigeneration Register, containing the Swedish population in families and spanning more than a century, and maintained as part of the Swedish Family-Cancer Database, which has been used in numerous previous studies in periodically updated versions.^23^ As family members, only first-degree relatives of offspring-parents and offspring and their siblings were considered. The offspring generation, for which siblings were available, reached a maximal age of 80 years by 2012; for the parental generation there was no age limits. Children of the offspring generation were not considered. Only the first AID diagnosis was included because multiple diagnoses may be liable to surveillance bias. Various revisions of the International Classification of Diseases codes were used for AIDs as described elsewhere.^24^ Cancers were retrieved from the Swedish Cancer Registry from years 1964–2012. The detailed SNOMED histology for specific types of NHL and HL was available from year 1993 onwards.

Person-years were calculated from the start of follow-up on 1 January 1964, until diagnosis of the relevant disease, death, emigration, or the end of the study (31 December 2012) whichever came first. SAS version 9.3 was used for the statistical analyses. Standardized incidence ratios (SIRs) were calculated for the offspring generation as the ratio of observed to expected number of cases. The expected numbers were calculated for all individuals without a history of an AID (when risk of AID was considered) or cancer (when risk of cancer was considered) and the rates were standardized by 5-year-age, gender, period (5 year group), socioeconomic status and residential area. The 95% confidence interval (95% CI) of the SIR was calculated assuming a Poisson distribution. All SIRs refer to the offspring generation. Results are considered significant when 95% CIs did not overlap with 1.00. The study was approved by the Ethical Committee of Lund University (2012/795).

## Results

Case numbers and diagnostic ages for the B-cell neoplasms and AIDs in the 0–80-year-old offspring generation are shown in Table 1. NHL was the most common cancer (15921 patients), followed by MM (4560) and HL (4434), male cases were more prevalent than female cases. Median diagnostic ages ranged from 29 years for HL to 61 for MM. Among 451133 AID patients, women were the majority and they were diagnosed at a higher median age (40 years) than men (37 years). Including also the parental generation, the total number of neoplasms was 64 418 (40 722 NHL, 6284 HL and 17 412 MM) and 531 155 AIDs.

Table 2 shows familial risks for B-cell neoplasms when family members were diagnosed with an AID. Only AID entries with at least 100 NHL cases or significant SIRs (increased or decreased, in any of the tables) are shown. SIRs by all 44 AIDs can be seen in Supplementary Table 1, which also lists the numbers of patients for each AID among offspring. The bottom line of Table 2 (‘All') gives the SIRs for cancer considering all AIDs; the SIR was increased for NHL (1.04), decreased for HL (0.93) and unchanged for MM (1.01). For NHL, significantly increased risks were noted for families of angiitis hypersensitive (3.58), Guillan-Barre (1.70), discoid lupus erythematosus (1.63), Sjogren (1.31) and psoriasis (1.08) patients. For HL, only families with pemphigus (3.88) showed an increased risk, whereas those with glomerular nephritis acute (0.38), ankylosing spondylitis (0.53) and Graves disease (0.75) had a decreased risk. For MM, no significant associations were noted. Among the three neoplasms, a total of six increased and three decreased associations with AIDs were observed.

Table 3 shows the risk for individual AIDs when family members were diagnose with a B-cell neoplasm. Detailed results with all 44 AIDs are shown in Supplementary Table 2. The overall SIRs were decreased for AID when family members were diagnosed with NHL (0.97) or MM (0.90). For NHL families, significantly increased risks were noted for families of angiitis hypersensitive (2.68), Sjogren (1.35), rheumatoid arthritis (1.08) and Wegener granulomatosis (1.55) patients. For psoriasis, the SIR was decreased to 0.90, opposite to Table 2. Decreased risks were noted also for Graves disease (0.87), Hashimoto thyroiditis (0.82) and ulcerative colitis (0.92). For HL families, Behcet disease (3.28), dermatitis herpetiformis (2.37), multiple sclerosis (1.27), primary biliary cirrhosis (2.01) and rheumatoid arthritis (1.24) risks were increased while celiac (0.72) and psoriasis (0.85) risks were decreased. For MM families, risks for celiac (0.72), type 1 diabetes (0.74), Graves disease (0.86), Hashimoto thyroiditis (0.73), psoriasis (0.84), systemic lupus erythematosus (0.72) and ulcerative colitis (0.85) were decreased, notably often matching with NHL. Considering all AIDs, the risk was increased for 9 AIDs and decreased for 13 AIDs by family history of B-cell neoplasm. Psoriasis risk was decreased by family history of all three B-cell neoplasms, and risks of celiac, Graves disease, Hashimoto thyroiditis and ulcerative colitis was decreased and the risk of rheumatoid arthritis was increased by family history of two B-cell neoplasms.

As many AIDs have sex preferences, we carried out a sex-specific analysis (no table shown). For NHL no AID was significantly associated when males or females were considered separately. For HL, the SIR was 11.07 (three cases, 95% CI: 2.09–32.77) when female relatives were diagnosed with pemphigus. Male MM was associated with angiitis hypersensitive in two cases (SIR: 20.92; 1.97–76.94), chronic rheumatic heart disease in 19 cases (1.79; 1.07–2.79) and primary biliary cirrhosis in four cases (4.66; 1.21–12.05). Female MM was associated with Graves disease in five cases (0.40; 0.12–0.93) and rheumatic fever in four cases (6.09; 1.58–15.74).

Data on specific subtypes of NHL and HL were available from year 1993 onwards (Table 4). For NHL, diffuse large B-cell lymphoma was associated with angiitis hypersensitive (8.67). The overall risk for diffuse large B-cell lymphoma with any AID in family members was 1.10 (869 cases, 1.03–1.18). The overall SIR for follicular lymphoma was not increased (1.01) but increased SIRs were noted when family members were diagnosed with discoid lupus erythematosus (3.11), glomerular nephritis chronic (1.71) and Guillan-Barre syndrome (2.75). For the rare mantel cell lymphoma, the SIR was 5.20 when family members were diagnosed with rheumatic fever.

For HL, the most common subtype nodular sclerosis classical HL, the SIR was 8.32 (2.17–21.53) for four cases with a family history of pemphigus. However, the overall SIR for nodular sclerosis classical HL was decreased to 0.81 (291; 0.72–0.90); the only significantly decreased association of 0.31 (0.06–0.91) was in three families with type 1 diabetes families.

## Discussion

The present exploratory data are novel in reporting familial risks between B-cell neoplasms and specific AIDs in two ways, for neoplasms by familial AIDs and for AIDs by familial neoplasms; the comparisons are independent for parent-offspring while some dependence may exist in sibling pairs. Only two such associations were found, NHL and angiitis hypersensitive and NHL and Sjogren syndrome. The first association was strongest for diffuse large B-cell lymphoma, whereas the second one appeared to be related to follicular lymphoma although the SIR was not significant. Thus a significant result in both of the two-way analyses is a strong evidence for a true association. The previously reported associations of HL with a family history of sarcoidosis (previous risk 1.8; 96% CI 1.01–3.1; present risk 1.16) and ulcerative colitis (previous risk 1.6; 1.02–2.6; present risk 1.00) and of MM with a family history of systemic lupus erythematosus (previous risk 2.66; 1.12–6.32; present risk 1.47) could not be replicated.^13, 15^ Although the Swedish population was partially overlapping in the previous and the present study, the current follow-up of cancer and AID was to 2012 instead of 1998 in the previous studies, and the present case numbers were doubled or trebled.^13, 15^

Considering the likelihood of false positives in the present study, the tested 44 AIDs and 3 neoplasms yielded 132 comparisons for each of Tables 2 and 3. Thus at 5% significance level, seven positive results could have been expected by chance. As nine positive associations were found for Table 2 and 22 for Table 3, the proportion of false positives is of concern. The larger number of positives in Table 3 is likely to be due to the larger sample size and statistical power because more familial cases were found for relatively young AID patients by old cancer patients than by the vice versa analysis in Table 2. Some features of the results support biological plausibility. First, of the 22 significant associations in Table 3, only 9 were found for a single AID-neoplasm pair and among these 9 two were supported by a similar observation in Table 2. Second, no significant opposite associations were found in Tables 2 and 3; if a family history of AID showed a decreased or increased association in Table 2, the risk for AID in Table 3 did not contradict the decreasing of increasing direction. The only exception was the NHL-psoriasis association (SIR 1.08 in Table 2 and 0.90 in Table 3).

The present results revealed large differences to those published on the risk of B-cell neoplasms subsequent to a personal AID also from the present Swedish population. The personal history was associated with a high overall risk of lymphoma (1.6 for NHL and 2.0 for HL) for any of 33 common AIDs and remarkably high risks after some AIDs, such as autoimmune hemolytic anemia (27 for NHL and 20 for HL); furthermore, a personal history was not associated with significantly decreased risks.^6, 7^ The personal history data on the current NHL–AID associations were available for discoid lupus erythematosus (current 1.63, personal history 2.7), Sjogren (1.31, 4.9) and psoriasis (1.08, 1.4). A personal history of AID did not influenced much the subsequent MM (overall SIR 1.1) but also for MM remarkably few negative associations were noted.^4^ The comparison of data from personal and family histories for lymphoma should be instructive and suggest mechanistic implications. As a personal history is a combination of a family history and a life-long history of somatic events, including immune challenges, it may not be surprising that the responses are higher after a personal history compared with a family history. An inherited gene variant, such as an human leukocyte antigen allele, may afford protection against lymphoma while a somatic immune dysregulation could plausible only increase risk through accumulation of genetic damage.

In conclusion, the present family data on B-cell neoplasms and 44 AIDs show an approximately equal number of associations for increase and decrease, suggesting that inherited genes or gene-environment interactions may increase the risk or be protective. In contrast, based on the Swedish literature, the associations in lymphoma through a positive individual history tend to only increase the risk, some severely, and the associated AIDs may be largely different from those found through a family history. For clinical practice, the take-home message could be that a family history of some AIDs my influence the risk of B-cell neoplasms but the magnitude of risk tends to be far lower than that found for a personal history of some AIDs.

## Figures and Tables

**Table 1:** Number of cases of NHL, HL, MM and autoimmune disorders in the study population of years 1964–2012

	*Men*	*Women*	*All*
*NHL*			
Cases	9583	6338	15921
Median age	56	57	56
Mean age	51.9±17.7	53.5±16.5	52.5±17.2
			
*HL*			
Cases	2543	1891	4434
Median age	30	28	29
Mean age	33.3±15.8	31.4±14.8	32.5±15.4
			
*MM*			
Cases	2698	1862	4560
Median age	61	62	61
Mean age	60.2±9.8	60.7±9.7	60.4±9.8
			
*Autoimmune disorders*			
Cases	189 246	261 887	451 133
Median age	37	40	39
Mean age	37.1±20.1	38.9±19.6	38.2±19.8

Abbreviations: HL, Hodgkin lymphoma; MM, multiple myeloma; NHL, non-Hodgkin lymphoma.

**Table 2:** SIRs of NHL, HL and MM by family history of autoimmune disorders for years 1964–2012

*Family history of AID (N of AID*)	*NHL*				*HL*				*MM*			
	*O*	*SIR*	*95% CI*		*O*	*SIR*	*95% CI*		*O*	*SIR*	*95% CI*	
Angiitis hypersensitive (130)	6	**3.58**	**1.29**	**7.84**	0	–	–	–	2	3.90	0.37	14.33
Ankylosing spondylitis (11227)	72	1.11	0.87	1.40	12	**0.53**	**0.27**	**0.93**	14	0.82	0.45	1.38
Behcet disease (500)	2	0.86	0.08	3.17	1	1.04	0.00	5.98	0	–	–	–
Celiac disease (35421)	82	1.02	0.81	1.27	22	0.70	0.44	1.07	20	1.02	0.62	1.57
Chronic rheumatic heart disease (3077)	128	0.89	0.74	1.05	35	1.19	0.83	1.66	52	1.13	0.85	1.49
Crohn disease (27659)	146	0.97	0.82	1.14	42	0.80	0.58	1.09	44	1.10	0.80	1.48
Dermatitis herpetiformis (1269)	15	1.09	0.61	1.79	8	2.22	0.95	4.40	1	0.24	0.00	1.38
Diabetes mellitus type I (26474)	29	1.07	0.72	1.54	13	0.70	0.37	1.20	1	0.28	0.00	1.62
Discoid lupus erythematosus (2067)	30	**1.63**	**1.10**	**2.33**	1	0.17	0.00	1.00	5	0.98	0.31	2.30
Glomerluar nephritis chronic (8405)	97	1.19	0.97	1.45	19	0.85	0.51	1.33	22	0.92	0.58	1.40
Glomerular nephritis acute (5957)	36	1.15	0.81	1.60	4	**0.38**	**0.10**	**0.99**	7	0.84	0.33	1.74
Graves disease (29016)	259	0.97	0.86	1.10	60	**0.75**	**0.58**	**0.97**	68	0.90	0.70	1.14
Guillain-Barre syndrome (2372)	35	**1.70**	**1.18**	**2.37**	4	0.68	0.18	1.75	4	0.67	0.17	1.73
Hashimoto thyroiditis (25614)	161	1.01	0.86	1.17	50	0.97	0.72	1.28	35	0.79	0.55	1.10
Multiple sclerosis (18200)	135	1.12	0.94	1.33	37	0.93	0.65	1.28	35	1.07	0.74	1.49
Pemphigus (644)	4	0.66	0.17	1.70	6	**3.88**	**1.40**	**8.51**	3	1.62	0.31	4.81
Polymyalgia rheumatica (7612)	269	1.04	0.92	1.18	62	1.15	0.88	1.48	85	1.04	0.83	1.28
Polymyositis/dermatomyositis (1384)	13	0.82	0.44	1.41	1	0.22	0.00	1.29	5	1.09	0.34	2.56
Psoriasis (92188)	630	**1.08**	**1.00**	**1.17**	170	0.89	0.76	1.03	155	0.97	0.83	1.14
Primary biliary cirrhosis (1696)	29	1.10	0.74	1.58	9	1.33	0.60	2.54	7	0.89	0.35	1.84
Rheumatoid arthritis (46277)	750	1.05	0.97	1.13	178	0.98	0.84	1.14	235	1.09	0.96	1.24
Sarcoidosis (13695)	112	0.98	0.81	1.18	39	1.16	0.82	1.58	34	1.05	0.73	1.47
Sjören syndrome (5755)	71	**1.31**	**1.03**	**1.66**	20	1.17	0.71	1.81	15	0.99	0.55	1.64
Systemic lupus erythematosus (5202)	55	1.11	0.84	1.45	14	0.99	0.54	1.66	21	1.47	0.91	2.26
Ulcerative colitis (46161)	271	1.00	0.89	1.13	89	1.00	0.80	1.23	70	0.95	0.74	1.20
Wegener granulomatosis (1517)	24	1.47	0.94	2.19	8	1.72	0.73	3.40	6	1.27	0.46	2.79
All (451133)	3893	**1.04**	**1.01**	**1.08**	1006	**0.93**	**0.87**	**0.99**	1079	1.01	0.95	1.07

Abbreviations: CI, confidence interval; HL, Hodgkin lymphoma; MM, multiple myeloma; NHL, non-Hodgkin lymphoma; O, observed cases; SIR, standardized incidence ratio. Bold type: 95% CI does not include 1.00.

**Table 3:** SIRs of autoimmune disorders by family history of NHL, HL and MM for years 1964–2012

*Autoimmune disorder in offspring*	*Family history of NHL*				*Family history of HL*				*Family history of MM*			
	*O*	*SIR*	*95% CI*		*O*	*SIR*	*95% CI*		*O*	*SIR*	*95% CI*	
Angiitis hypersensitive	7	**2.68**	**1.06**	**5.55**	1	2.47	0.00	14.17	2	1.68	0.16	6.17
Ankylosing spondylitis	255	1.00	0.88	1.13	31	0.84	0.57	1.19	109	1.00	0.83	1.21
Behcet disease	7	0.70	0.28	1.46	5	**3.28**	**1.04**	**7.72**	2	0.50	0.05	1.82
Celiac disease	364	0.92	0.83	1.02	59	**0.72**	**0.54**	**0.92**	107	**0.72**	**0.59**	**0.87**
Chronic rheumatic heart disease	64	0.86	0.67	1.10	13	1.28	0.68	2.19	33	0.99	0.68	1.39
Crohn disease	507	0.92	0.84	1.01	84	0.99	0.79	1.22	206	0.91	0.79	1.04
Dermatitis herpetiformis	38	1.26	0.89	1.73	10	**2.37**	**1.13**	**4.38**	13	0.95	0.50	1.63
Diabetes mellitus type I	231	0.96	0.84	1.09	51	0.87	0.64	1.14	57	**0.74**	**0.56**	**0.96**
Discoid lupus erythematosus	59	1.18	0.90	1.52	8	1.14	0.49	2.25	25	1.11	0.72	1.65
Glomerular nephritis chronic	178	0.93	0.80	1.08	28	1.02	0.68	1.48	77	0.94	0.74	1.18
Glomerular nephritis acute	103	1.05	0.86	1.27	16	0.96	0.55	1.56	36	0.92	0.65	1.28
Graves disease	586	**0.87**	**0.80**	**0.94**	91	0.95	0.76	1.16	249	**0.86**	**0.75**	**0.97**
Guillain-Barre syndrome	60	1.13	0.86	1.45	6	0.79	0.28	1.72	20	0.86	0.53	1.33
Hashimoto thyroiditis	424	**0.82**	**0.75**	**0.91**	78	1.00	0.79	1.25	158	**0.73**	**0.62**	**0.86**
Multiple sclerosis	465	1.09	0.99	1.19	77	**1.27**	**1.00**	**1.59**	177	0.96	0.83	1.12
Pemphigus	12	1.11	0.57	1.95	5	2.84	0.90	6.69	7	1.53	0.61	3.16
Polymyalgia rheumatica	202	0.98	0.85	1.13	36	1.34	0.94	1.85	108	1.09	0.89	1.31
Polymyositis/dermatomyositis	35	1.10	0.77	1.54	4	0.89	0.23	2.31	11	0.78	0.39	1.39
Primary biliary cirrhosis	53	1.19	0.89	1.56	12	**2.01**	**1.04**	**3.53**	20	0.97	0.59	1.50
Psoriasis	1884	**0.90**	**0.86**	**0.94**	258	**0.85**	**0.75**	**0.97**	776	**0.84**	**0.79**	**0.91**
Rheumatoid arthritis	1236	**1.08**	**1.02**	**1.14**	197	**1.24**	**1.08**	**1.43**	507	0.98	0.89	1.06
Sarcoidosis	348	1.04	0.94	1.16	50	1.07	0.79	1.41	151	1.04	0.88	1.22
Sjören syndrome	200	**1.35**	**1.17**	**1.55**	24	1.20	0.76	1.78	77	1.13	0.89	1.41
Systemic lupus erythematosus	133	1.14	0.96	1.35	15	0.88	0.49	1.46	36	**0.72**	**0.50**	**0.99**
Ulcerative colitis	892	**0.92**	**0.86**	**0.98**	170	1.16	0.99	1.35	346	**0.85**	**0.76**	**0.95**
Wegener granulomatosis	57	**1.55**	**1.17**	**2.01**	5	0.97	0.31	2.29	21	1.25	0.77	1.91
All	9108	**0.97**	**0.95**	**0.99**	1437	1.02	0.97	1.07	3631	**0.90**	**0.87**	**0.93**

Abbreviations: CI, confidence interval; HL, Hodgkin lymphoma; MM, multiple myeloma; NHL, non-Hodgkin lymphoma; O, observed cases; SIR, standardized incidence ratio.

Bold type: 95% CI does not include 1.00.

**Table 4:** SIRs of subtypes of NHL by family history of autoimmune disorders for years 1993–2012

*Family history of autoimmune disorder*	*Diffuse large B cell*				*Follicular*				*Mantel cell*			
	*O*	*SIR*	*95% CI*		*O*	*SIR*	*95% CI*		*O*	*SIR*	*95% CI*	
Angiitis hypersensitive	3	**8.67**	**1.64**	**25.67**	1	3.71	0.00	21.27	0			
Ankylosing spondylitis	13	0.95	0.50	1.63	13	1.32	0.70	2.26	6	2.56	0.92	5.61
Behcet disease	0				1	2.85	0.00	16.36	0			
Celiac disease	20	1.21	0.74	1.88	12	1.04	0.54	1.83	1	0.36	0.00	2.09
Chronic rheumatic heart disease	25	0.82	0.53	1.22	19	0.81	0.48	1.26	9	1.47	0.67	2.81
Crohn disease	35	1.10	0.76	1.53	21	0.90	0.56	1.38	9	1.63	0.74	3.12
Dermatitis herpetiformis	5	1.70	0.54	4.01	0				1	1.74	0.00	9.96
Diabetes mellitus type I	7	1.28	0.51	2.65	2	0.62	0.06	2.29	0			
Discoid lupus erythematosus	5	1.28	0.40	3.01	9	**3.11**	**1.41**	**5.94**	1	1.44	0.00	8.28
Glomerluar nephritis chronic	18	1.05	0.62	1.66	22	**1.72**	**1.08**	**2.61**	3	0.94	0.18	2.77
Glomerular nephritis acute	7	1.07	0.42	2.21	4	0.84	0.22	2.16	2	1.77	0.17	6.50
Graves disease	58	1.04	0.79	1.34	38	0.91	0.64	1.25	9	0.87	0.40	1.66
Guillain-Barre syndrome	5	1.16	0.37	2.72	9	**2.75**	**1.25**	**5.25**	3	3.70	0.70	10.96
Hashimoto thyroiditis	41	1.21	0.87	1.65	21	0.85	0.53	1.30	5	0.83	0.26	1.95
Multiple sclerosis	35	1.38	0.96	1.92	16	0.86	0.49	1.39	4	0.89	0.23	2.30
Pemphigus	0				2	2.06	0.19	7.59	0			
Polymyalgia rheumatica	64	1.18	0.91	1.50	35	0.84	0.58	1.16	11	0.99	0.49	1.77
Polymyositis/dermatomyositis	7	2.08	0.83	4.31	3	1.21	0.23	3.58	0			
Psoriasis	140	1.13	0.95	1.34	95	1.05	0.85	1.28	17	0.78	0.45	1.25
Rheumatic fever	6	1.20	0.43	2.63	2	0.52	0.05	1.91	5	**5.20**	**1.64**	**12.24**
Rheumatoid arthritis	164	1.09	0.93	1.27	132	1.15	0.97	1.37	21	0.73	0.45	1.11
Sarcoidosis	26	1.08	0.70	1.58	15	0.85	0.47	1.40	2	0.45	0.04	1.66
Sjören syndrome	13	1.13	0.60	1.94	14	1.64	0.89	2.76	3	1.44	0.27	4.27
Systemic lupus erythematosus	15	1.44	0.81	2.39	3	0.38	0.07	1.13	1	0.52	0.00	2.97
Ulcerative colitis	52	0.91	0.68	1.19	39	0.93	0.66	1.27	10	0.99	0.47	1.83
Wegener granulomatosis	8	2.29	0.98	4.54	3	1.12	0.21	3.33	0			
All	869	**1.10**	**1.03**	**1.18**	592	1.01	0.93	1.09	136	0.93	0.78	1.10

Abbreviations: CI, confidence interval; O, observed cases; SIR, standardized incidence ratio.

Bold type: 95% CI does not include 1.00.
