# Supplementary material for: Familial associations of lymphoma and myeloma with autoimmune diseases
Source: Blood Cancer J. 2017 Jan 6;7(1):e515–. doi: 10.1038/bcj.2016.123 (PMC5301032; doi:10.1038/bcj.2016.123)
Supplement: Supplementary Tables [file bcj2016123x1.docx]

**FAMILIAL ASSOCIATIONS OF LYMPHOMA AND MYELOMA WITH AUTOIMMUNE DISEASES**

Kari Hemminki ^1,2^, Asta Försti^1,2^, Kristina Sundquist^2,3^ , Jan Sundquist^2,3^ and Xinjun Li^2^

^1^ Division of Molecular Genetic Epidemiology, German Cancer Research Center (DKFZ), Im Neuenheimer Feld 580, D-69120, Heidelberg, Germany

^2^ Center for Primary Health Care Research, Lund University, SE-205 02 Malmö, Sweden

^3^ Stanford Prevention Research Center, Stanford University School of Medicine, Stanford, California 94305-5705, USA

Correspondence: Kari Hemminki, Division of Molecular Genetic Epidemiology, German Cancer Research Center (DKFZ), Im Neuenheimer Feld 580, D-69120 Heidelberg, Germany.

Telephone:  +496221421800
Fax: +496221421810

Email: kari.hemminki@dkfz.de

Word count: 2099 (text), 199 (abstract).

Running title: blood cancer and autoimmune diseases

| **Supplementary Table 1. SIRs of HL, NHL, and MM by family history of autoimmune disorders** | | | | | | | | | | | | | | | | | | | | | | | | | | | | | |
| --- | --- | --- | --- | --- | --- | --- | --- | --- | --- | --- | --- | --- | --- | --- | --- | --- | --- | --- | --- | --- | --- | --- | --- | --- | --- | --- | --- | --- | --- |
|  | | NHL | | | | | | | |  | | HL | | | | | | | |  | | MM | | | | | | | |
| Family history of AID (N=AIDs) | | O | | SIR | | 95% CI | | | |  | | O | | SIR | | 95% CI | | | |  | | O | | SIR | | 95% CI | | | |
| Addison disease (1736) | | 16 | | 1.17 | | 0.67 | | 1.90 | |  | | 4 | | 0.96 | | 0.25 | | 2.49 | |  | | 6 | | 1.55 | | 0.56 | | 3.40 | |
| Amyotrophic lateral sclerosis (2952) | | 71 | | 1.12 | | 0.87 | | 1.41 | |  | | 15 | | 0.98 | | 0.55 | | 1.63 | |  | | 20 | | 1.03 | | 0.63 | | 1.59 | |
| Angiitis hypersensitive (130) | | 6 | | **3.58** | | **1.29** | | **7.84** | |  | | 0 | |  | |  | |  | |  | | 2 | | 3.90 | | 0.37 | | 14.33 | |
| Ankylosing spondylitis (11227) | | 72 | | 1.11 | | 0.87 | | 1.40 | |  | | 12 | | **0.53** | | **0.27** | | **0.93** | |  | | 14 | | 0.82 | | 0.45 | | 1.38 | |
| Autoimmune hemolytic anemia (360) | | 6 | | 0.79 | | 0.28 | | 1.73 | |  | | 2 | | 1.24 | | 0.12 | | 4.56 | |  | | 1 | | 0.42 | | 0.00 | | 2.40 | |
| Behcet disease (500) | | 2 | | 0.86 | | 0.08 | | 3.17 | |  | | 1 | | 1.04 | | 0.00 | | 5.98 | |  | | 0 | |  | |  | |  | |
| Celiac disease (35421) | | 82 | | 1.02 | | 0.81 | | 1.27 | |  | | 22 | | 0.70 | | 0.44 | | 1.07 | |  | | 20 | | 1.02 | | 0.62 | | 1.57 | |
| Chorea minor (75) | | 0 | |  | |  | |  | |  | | 1 | | 5.73 | | 0.00 | | 32.83 | |  | | 0 | |  | |  | |  | |
| Chronic rheumatic heart disease (3077) | | 128 | | 0.89 | | 0.74 | | 1.05 | |  | | 35 | | 1.19 | | 0.83 | | 1.66 | |  | | 52 | | 1.13 | | 0.85 | | 1.49 | |
| Crohn disease (27659) | | 146 | | 0.97 | | 0.82 | | 1.14 | |  | | 42 | | 0.80 | | 0.58 | | 1.09 | |  | | 44 | | 1.10 | | 0.80 | | 1.48 | |
| Dermatitis Herpetiformis (1269) | | 15 | | 1.09 | | 0.61 | | 1.79 | |  | | 8 | | 2.22 | | 0.95 | | 4.40 | |  | | 1 | | 0.24 | | 0.00 | | 1.38 | |
| Diabetes mellitus type I (26474) | | 29 | | 1.07 | | 0.72 | | 1.54 | |  | | 13 | | 0.70 | | 0.37 | | 1.20 | |  | | 1 | | 0.28 | | 0.00 | | 1.62 | |
| Discoid lupus erythematosus (2067) | | 30 | | **1.63** | | **1.10** | | **2.33** | |  | | 1 | | 0.17 | | 0.00 | | 1.00 | |  | | 5 | | 0.98 | | 0.31 | | 2.30 | |
| Giant-cell arteritis (4696) | | 77 | | 0.91 | | 0.72 | | 1.14 | |  | | 16 | | 0.75 | | 0.43 | | 1.22 | |  | | 30 | | 1.18 | | 0.79 | | 1.68 | |
| Glomerluar nephritis chronic (8405) | | 97 | | 1.19 | | 0.97 | | 1.45 | |  | | 19 | | 0.85 | | 0.51 | | 1.33 | |  | | 22 | | 0.92 | | 0.58 | | 1.40 | |
| Glomerular nephritis acute (5957) | | 36 | | 1.15 | | 0.81 | | 1.60 | |  | | 4 | | **0.38** | | **0.10** | | **0.99** | |  | | 7 | | 0.84 | | 0.33 | | 1.74 | |
| Graves disease (29016) | | 259 | | 0.97 | | 0.86 | | 1.10 | |  | | 60 | | **0.75** | | **0.58** | | **0.97** | |  | | 68 | | 0.90 | | 0.70 | | 1.14 | |
| Guillain-Barre Syndrome (2372) | | 35 | | **1.70** | | **1.18** | | **2.37** | |  | | 4 | | 0.68 | | 0.18 | | 1.75 | |  | | 4 | | 0.67 | | 0.17 | | 1.73 | |
| Hashimoto thyroiditis (25614) | | 161 | | 1.01 | | 0.86 | | 1.17 | |  | | 50 | | 0.97 | | 0.72 | | 1.28 | |  | | 35 | | 0.79 | | 0.55 | | 1.10 | |
| Immune thrombocytopenic purpura (7068) | | 38 | | 0.91 | | 0.65 | | 1.25 | |  | | 16 | | 1.23 | | 0.70 | | 2.00 | |  | | 12 | | 1.04 | | 0.53 | | 1.82 | |
| Localized scleroderma (1844) | | 10 | | 0.86 | | 0.41 | | 1.58 | |  | | 0 | | 0.00 | | 0.26 | | 1.06 | |  | | 2 | | 0.62 | | 0.06 | | 2.28 | |
| Lupoid hepatitis (3275) | | 35 | | 1.09 | | 0.76 | | 1.51 | |  | | 12 | | 1.26 | | 0.65 | | 2.20 | |  | | 9 | | 0.99 | | 0.45 | | 1.88 | |
| Multiple sclerosis (18200) | | 135 | | 1.12 | | 0.94 | | 1.33 | |  | | 37 | | 0.93 | | 0.65 | | 1.28 | |  | | 35 | | 1.07 | | 0.74 | | 1.49 | |
| Myasthenia gravis (1898) | | 23 | | 1.02 | | 0.65 | | 1.53 | |  | | 6 | | 0.97 | | 0.35 | | 2.13 | |  | | 6 | | 0.91 | | 0.33 | | 1.99 | |
| Pemphigoid (1005) | | 29 | | 0.78 | | 0.52 | | 1.11 | |  | | 5 | | 0.63 | | 0.20 | | 1.48 | |  | | 13 | | 1.11 | | 0.59 | | 1.90 | |
| Pemphigus (644) | | 4 | | 0.66 | | 0.17 | | 1.70 | |  | | 6 | | **3.88** | | **1.40** | | **8.51** | |  | | 3 | | 1.62 | | 0.31 | | 4.81 | |
| Pernicious anemia (938) | | 59 | | 0.94 | | 0.72 | | 1.22 | |  | | 8 | | 0.80 | | 0.34 | | 1.59 | |  | | 18 | | 0.85 | | 0.50 | | 1.35 | |
| Polyarteritis nodosa (567) | | 10 | | 1.05 | | 0.50 | | 1.95 | |  | | 3 | | 1.33 | | 0.25 | | 3.94 | |  | | 1 | | 0.35 | | 0.00 | | 1.98 | |
| Polymyalgia rheumatica (7612) | | 269 | | 1.04 | | 0.92 | | 1.18 | |  | | 62 | | 1.15 | | 0.88 | | 1.48 | |  | | 85 | | 1.04 | | 0.83 | | 1.28 | |
| Polymyositis/dermatomyositis (1384) | | 13 | | 0.82 | | 0.44 | | 1.41 | |  | | 1 | | 0.22 | | 0.00 | | 1.29 | |  | | 5 | | 1.09 | | 0.34 | | 2.56 | |
| Primary biliary cirrhosis (1696) | | 29 | | 1.10 | | 0.74 | | 1.58 | |  | | 9 | | 1.33 | | 0.60 | | 2.54 | |  | | 7 | | 0.89 | | 0.35 | | 1.84 | |
| Psoriasis (92188) | | 630 | | **1.08** | | **1.00** | | **1.17** | |  | | 170 | | 0.89 | | 0.76 | | 1.03 | |  | | 155 | | 0.97 | | 0.83 | | 1.14 | |
| Reiter disease (1098) | | 8 | | 0.98 | | 0.42 | | 1.93 | |  | | 1 | | 0.38 | | 0.00 | | 2.20 | |  | | 2 | | 0.87 | | 0.08 | | 3.21 | |
| Rheumatic fever (1918) | | 28 | | 1.18 | | 0.78 | | 1.70 | |  | | 4 | | 0.64 | | 0.17 | | 1.66 | |  | | 8 | | 1.13 | | 0.48 | | 2.24 | |
| Rheumatoid arthritis (46277) | | 750 | | 1.05 | | 0.97 | | 1.13 | |  | | 178 | | 0.98 | | 0.84 | | 1.14 | |  | | 235 | | 1.09 | | 0.96 | | 1.24 | |
| Sarcoidosis (13695) | | 112 | | 0.98 | | 0.81 | | 1.18 | |  | | 39 | | 1.16 | | 0.82 | | 1.58 | |  | | 34 | | 1.05 | | 0.73 | | 1.47 | |
| Sjören syndrome (5755) | | 71 | | **1.31** | | **1.03** | | **1.66** | |  | | 20 | | 1.17 | | 0.71 | | 1.81 | |  | | 15 | | 0.99 | | 0.55 | | 1.64 | |
| Systemic lupus erythematosus (5202) | | 55 | | 1.11 | | 0.84 | | 1.45 | |  | | 14 | | 0.99 | | 0.54 | | 1.66 | |  | | 21 | | 1.47 | | 0.91 | | 2.26 | |
| Systemic sclerosis (1725) | | 19 | | 0.93 | | 0.56 | | 1.46 | |  | | 9 | | 1.57 | | 0.71 | | 2.99 | |  | | 5 | | 0.84 | | 0.27 | | 1.98 | |
| Takayasus disease (209) | | 2 | | 1.22 | | 0.12 | | 4.49 | |  | | 0 | |  | |  | |  | |  | | 0 | |  | |  | |  | |
| Thrombotic thrombocytop (250) | | 1 | | 0.48 | | 0.00 | | 2.73 | |  | | 0 | |  | |  | |  | |  | | 0 | |  | |  | |  | |
| Ulcerative colitis (46161) | | 271 | | 1.00 | | 0.89 | | 1.13 | |  | | 89 | | 1.00 | | 0.80 | | 1.23 | |  | | 70 | | 0.95 | | 0.74 | | 1.20 | |
| Wegener granulomatosis (1517) | | 24 | | 1.47 | | 0.94 | | 2.19 | |  | | 8 | | 1.72 | | 0.73 | | 3.40 | |  | | 6 | | 1.27 | | 0.46 | | 2.79 | |
| All (451133) | | 3893 | | **1.04** | | **1.01** | | **1.08** | |  | | 1006 | | **0.93** | | **0.87** | | **0.99** | |  | | 1079 | | 1.01 | | 0.95 | | 1.07 | |
| O = Observed cases; SIR = Standardized incidence ratio; CI = Confidence interval. | | | | | | | | | | | | | | | | | | | | | | | | | | | | | |
| Bold type: 95% confidence interval does not include 1.00. | | | | | | | |  | |  | |  | |  | |  | |  | |  | |  | |  | |  | |  | |
| **Supplementary Table 2. SIRs of autoimmune disorders by family history of HL, NHL, and MM** | | | | | | | | | | | | | | | | | | | | | | | | | | | | |  |
|  | Family history of NHL | | | | | | | |  | | Family history of HL | | | | | | | |  | | Family history of MM | | | | | | | |  |
| Autoimmune disorder in offspring | O | | SIR | | 95% CI | | | |  | | O | | SIR | | 95% CI | | | |  | | O | | SIR | | 95% CI | | | |  |
| Addison disease | 32 | | 0.86 | | 0.59 | | 1.21 | |  | | 6 | | 1.09 | | 0.39 | | 2.38 | |  | | 20 | | 1.28 | | 0.78 | | 1.98 | |  |
| Amyotrophic lateral sclerosis | 85 | | 1.16 | | 0.93 | | 1.43 | |  | | 7 | | 0.70 | | 0.28 | | 1.44 | |  | | 29 | | 0.85 | | 0.57 | | 1.22 | |  |
| Angiitis hypersensitive | 7 | | **2.68** | | **1.06** | | **5.55** | |  | | 1 | | 2.47 | | 0.00 | | 14.17 | |  | | 2 | | 1.68 | | 0.16 | | 6.17 | |  |
| Ankylosing spondylitis | 255 | | 1.00 | | 0.88 | | 1.13 | |  | | 31 | | 0.84 | | 0.57 | | 1.19 | |  | | 109 | | 1.00 | | 0.83 | | 1.21 | |  |
| Autoimmune hemolytic anemia | 8 | | 1.23 | | 0.53 | | 2.44 | |  | | 1 | | 0.95 | | 0.00 | | 5.45 | |  | | 2 | | 0.74 | | 0.07 | | 2.70 | |  |
| Behcet disease | 7 | | 0.70 | | 0.28 | | 1.46 | |  | | 5 | | **3.28** | | **1.04** | | **7.72** | |  | | 2 | | 0.50 | | 0.05 | | 1.82 | |  |
| Celiac disease | 364 | | 0.92 | | 0.83 | | 1.02 | |  | | 59 | | **0.72** | | **0.54** | | **0.92** | |  | | 107 | | **0.72** | | **0.59** | | **0.87** | |  |
| Chorea minor | 0 | |  | |  | |  | |  | | 0 | |  | |  | |  | |  | | 1 | | 2.40 | | 0.00 | | 13.74 | |  |
| Chronic rheumatic heart disease | 64 | | 0.86 | | 0.67 | | 1.10 | |  | | 13 | | 1.28 | | 0.68 | | 2.19 | |  | | 33 | | 0.99 | | 0.68 | | 1.39 | |  |
| Crohn disease | 507 | | 0.92 | | 0.84 | | 1.01 | |  | | 84 | | 0.99 | | 0.79 | | 1.22 | |  | | 206 | | 0.91 | | 0.79 | | 1.04 | |  |
| Dermatitis Herpetiformis | 38 | | 1.26 | | 0.89 | | 1.73 | |  | | 10 | | **2.37** | | **1.13** | | **4.38** | |  | | 13 | | 0.95 | | 0.50 | | 1.63 | |  |
| Diabetes mellitus type I | 231 | | 0.96 | | 0.84 | | 1.09 | |  | | 51 | | 0.87 | | 0.64 | | 1.14 | |  | | 57 | | **0.74** | | **0.56** | | **0.96** | |  |
| Discoid lupus erythematosus | 59 | | 1.18 | | 0.90 | | 1.52 | |  | | 8 | | 1.14 | | 0.49 | | 2.25 | |  | | 25 | | 1.11 | | 0.72 | | 1.65 | |  |
| Giant-cell arteritis | 120 | | 0.95 | | 0.78 | | 1.13 | |  | | 19 | | 1.15 | | 0.69 | | 1.80 | |  | | 60 | | 0.98 | | 0.75 | | 1.26 | |  |
| Glomerluar nephritis chronic | 178 | | 0.93 | | 0.80 | | 1.08 | |  | | 28 | | 1.02 | | 0.68 | | 1.48 | |  | | 77 | | 0.94 | | 0.74 | | 1.18 | |  |
| Glomerular nephritis acute | 103 | | 1.05 | | 0.86 | | 1.27 | |  | | 16 | | 0.96 | | 0.55 | | 1.56 | |  | | 36 | | 0.92 | | 0.65 | | 1.28 | |  |
| Graves disease | 586 | | **0.87** | | **0.80** | | **0.94** | |  | | 91 | | 0.95 | | 0.76 | | 1.16 | |  | | 249 | | **0.86** | | **0.75** | | **0.97** | |  |
| Guillain-Barre Syndrome | 60 | | 1.13 | | 0.86 | | 1.45 | |  | | 6 | | 0.79 | | 0.28 | | 1.72 | |  | | 20 | | 0.86 | | 0.53 | | 1.33 | |  |
| Hashimoto thyroiditis | 424 | | **0.82** | | **0.75** | | **0.91** | |  | | 78 | | 1.00 | | 0.79 | | 1.25 | |  | | 158 | | **0.73** | | **0.62** | | **0.86** | |  |
| Immune thrombocytopenic purpura | 111 | | 1.05 | | 0.86 | | 1.26 | |  | | 23 | | 1.22 | | 0.78 | | 1.84 | |  | | 38 | | 0.89 | | 0.63 | | 1.23 | |  |
| Localized scleroderma | 30 | | 0.81 | | 0.55 | | 1.16 | |  | | 4 | | 0.71 | | 0.18 | | 1.84 | |  | | 11 | | 0.70 | | 0.35 | | 1.25 | |  |
| Lupoid hepatitis | 77 | | 1.04 | | 0.82 | | 1.30 | |  | | 10 | | 0.93 | | 0.44 | | 1.72 | |  | | 35 | | 1.11 | | 0.77 | | 1.54 | |  |
| Multiple sclerosis | 465 | | 1.09 | | 0.99 | | 1.19 | |  | | 77 | | **1.27** | | **1.00** | | **1.59** | |  | | 177 | | 0.96 | | 0.83 | | 1.12 | |  |
| Myasthenia gravis | 41 | | 0.93 | | 0.67 | | 1.26 | |  | | 10 | | 1.62 | | 0.77 | | 2.99 | |  | | 15 | | 0.77 | | 0.43 | | 1.28 | |  |
| Pemphigoid | 22 | | 0.88 | | 0.55 | | 1.33 | |  | | 3 | | 0.89 | | 0.17 | | 2.63 | |  | | 10 | | 0.85 | | 0.40 | | 1.56 | |  |
| Pemphigus | 12 | | 1.11 | | 0.57 | | 1.95 | |  | | 5 | | 2.84 | | 0.90 | | 6.69 | |  | | 7 | | 1.53 | | 0.61 | | 3.16 | |  |
| Pernicious anemia | 25 | | 1.10 | | 0.71 | | 1.62 | |  | | 3 | | 0.95 | | 0.18 | | 2.82 | |  | | 8 | | 0.78 | | 0.33 | | 1.55 | |  |
| Polyarteritis nodosa | 11 | | 0.93 | | 0.46 | | 1.68 | |  | | 0 | |  | |  | |  | |  | | 7 | | 1.38 | | 0.55 | | 2.86 | |  |
| Polymyalgia rheumatica | 202 | | 0.98 | | 0.85 | | 1.13 | |  | | 36 | | 1.34 | | 0.94 | | 1.85 | |  | | 108 | | 1.09 | | 0.89 | | 1.31 | |  |
| Polymyositis/dermatomyositis | 35 | | 1.10 | | 0.77 | | 1.54 | |  | | 4 | | 0.89 | | 0.23 | | 2.31 | |  | | 11 | | 0.78 | | 0.39 | | 1.39 | |  |
| Primary biliary cirrhosis | 53 | | 1.19 | | 0.89 | | 1.56 | |  | | 12 | | **2.01** | | **1.04** | | **3.53** | |  | | 20 | | 0.97 | | 0.59 | | 1.50 | |  |
| Psoriasis | 1884 | | **0.90** | | **0.86** | | **0.94** | |  | | 258 | | **0.85** | | **0.75** | | **0.97** | |  | | 776 | | **0.84** | | **0.79** | | **0.91** | |  |
| Reiter disease | 28 | | 1.07 | | 0.71 | | 1.55 | |  | | 3 | | 0.82 | | 0.16 | | 2.44 | |  | | 12 | | 1.06 | | 0.55 | | 1.86 | |  |
| Rheumatic fever | 52 | | 1.16 | | 0.87 | | 1.53 | |  | | 3 | | 0.48 | | 0.09 | | 1.42 | |  | | 26 | | 1.37 | | 0.89 | | 2.01 | |  |
| Rheumatoid arthritis | 1236 | | **1.08** | | **1.02** | | **1.14** | |  | | 197 | | **1.24** | | **1.08** | | **1.43** | |  | | 507 | | 0.98 | | 0.89 | | 1.06 | |  |
| Sarcoidosis | 348 | | 1.04 | | 0.94 | | 1.16 | |  | | 50 | | 1.07 | | 0.79 | | 1.41 | |  | | 151 | | 1.04 | | 0.88 | | 1.22 | |  |
| Sjören syndrome | 200 | | **1.35** | | **1.17** | | **1.55** | |  | | 24 | | 1.20 | | 0.76 | | 1.78 | |  | | 77 | | 1.13 | | 0.89 | | 1.41 | |  |
| Systemic lupus erythematosus | 133 | | 1.14 | | 0.96 | | 1.35 | |  | | 15 | | 0.88 | | 0.49 | | 1.46 | |  | | 36 | | **0.72** | | **0.50** | | **0.99** | |  |
| Systemic sclerosis | 54 | | 1.29 | | 0.97 | | 1.69 | |  | | 11 | | 1.90 | | 0.94 | | 3.40 | |  | | 22 | | 1.17 | | 0.73 | | 1.77 | |  |
| Takayasus disease | 5 | | 1.06 | | 0.33 | | 2.49 | |  | | 0 | |  | |  | |  | |  | | 2 | | 0.99 | | 0.09 | | 3.62 | |  |
| Thrombotic thrombocytop | 7 | | 1.39 | | 0.55 | | 2.87 | |  | | 0 | |  | |  | |  | |  | | 2 | | 0.90 | | 0.08 | | 3.30 | |  |
| Ulcerative colitis | 892 | | **0.92** | | **0.86** | | **0.98** | |  | | 170 | | 1.16 | | 0.99 | | 1.35 | |  | | 346 | | **0.85** | | **0.76** | | **0.95** | |  |
| Wegener granulomatosis | 57 | | **1.55** | | **1.17** | | **2.01** | |  | | 5 | | 0.97 | | 0.31 | | 2.29 | |  | | 21 | | 1.25 | | 0.77 | | 1.91 | |  |
| All | 9108 | | **0.97** | | **0.95** | | **0.99** | |  | | 1437 | | 1.02 | | 0.97 | | 1.07 | |  | | 3631 | | **0.90** | | **0.87** | | **0.93** | |  |
| O = Observed cases; SIR = Standardized incidence ratio; CI = Confidence interval. | | | | | | | | | | | | | | | | | | |  | |  | |  | |  | |  | |  |
| Bold type: 95% confidence interval does not include 1.00. | | | | | | | | |  | |  | |  | |  | |  | |  | |  | |  | |  | |  | |  |
